# Recent advances in managing and understanding Klinefelter syndrome

**DOI:** 10.12688/f1000research.16747.1

**Published:** 2019-01-28

**Authors:** Priyanka Bearelly, Robert Oates

**Affiliations:** 1 Urology, Boston University School of Medicine, 725 Albany Street, Suite 3B, Boston, MA, 02118, USA

**Keywords:** Klinefelter Syndrome, azoospermia, testis, TESE, 47, XXY

## Abstract

Klinefelter syndrome can present as a wide spectrum of clinical manifestations at various stages in life, making it a chromosomal disorder with no standardized set of guidelines for appropriate management. Understanding the genetic and hormonal causes of this syndrome can allow physicians to treat each patient on a more individualized basis. The timing of diagnosis and degree of symptoms can guide management. This report will provide an updated review of the clinical presentation at various stages in life and the implications for management.

## Introduction

Klinefelter syndrome (KS) is a common male chromosomal disorder (47, XXY) that has been a topic of intrigue and inquiry since the 1940s, when it was first described ^1^. It is a subject of interest because of its wide spectrum of clinical manifestations, which include certain physical features, cognitive delays, and azoospermia.

The chromosomal aberration found in KS is due to either meiotic or mitotic non-disjunction, leading to sex-chromosomal aneuploidy. Two genetic variations exist. The majority (90%) of cases present as a pure form with a 47, XXY karyotype, whereas the remaining 10% include the following sex-chromosomal abnormalities: mosaicism (46, XY/47, XXY), higher-grade aneuploidy (48, XXXY; 49, XXXXY), and structurally abnormal X chromosomes ^2^.

KS has been reported to occur in 1 out of 600 male births, approximately 64% of which remain undiagnosed throughout life ^3, 4^. However, KS may be recognized during the prenatal, prepubertal, adolescent, or adult period. A patient’s management is guided by the extent and severity of clinical features as well as timing of presentation.

## How does management differ according to time of diagnosis?

There is no single, classic phenotype that can describe KS. The familiar description of tall stature with a thin, eunuchoid body habitus is not only inaccurate but also inadequate. There are no distinct dysmorphic features, and presentation may vary according to the degree of gonadal dysfunction ^5^. The severity of the presentation is also strongly correlated with the severity of the sex-chromosome aneuploidy (that is, higher-grade aneuploidies). A prepubertal boy may be diagnosed during the workup of cryptorchidism or mild developmental delay, whereas KS may be discovered initially in the older individual during an infertility evaluation. Some physicians believe that delay in diagnosis can increase the morbidity of the patient; therefore, it is important for practitioners to become familiar with this spectrum of features.

There are no telltale physical signs of KS at birth. An increasingly important means of diagnosis is through prenatal testing. There is a growing utilization of non-invasive prenatal screening by cell-free fetal DNA testing. This test can identify the presence of extra chromosomes—autosomal and sex ^6^. However, Nieschlag, Ferlin, Gravholt *et al*. provided a summary of the Second International Workshop on Klinefelter Syndrome (Münster, Germany, in 2016), and one of the topics addressed was the advisability of neonatal screening and diagnosis ^7^. Whereas Rogol argued that there are recognizable benefits to neonatal detection, Gravholt cautioned that there is yet no proof that early detection necessarily results in a reduction in morbidity and mortality with an improvement in outcomes when large populations are screened. Therefore, even expert opinion diverges on this issue at this time. The mini-puberty, namely activated luteinizing hormone (LH) and follicle-stimulating hormone (FSH) release in the first few months of life, matches that of 46, XY infants. Less than 3% to 10% of patients are diagnosed during the prepubertal period ^4^. Tall stature may be seen, but not always, because of increased dosage of the sex chromosome-related short stature homeobox-containing gene ( *SHOX*), which resides in the pseudoautosomal region of both X and Y and is not subject to X-chromosomal inactivation ^8^. Additional physical features include cryptorchidism, gynecomastia, hypotonia, hypertelorism, clinodactyly, elbow dysplasia, pes planus, and a high arched palate ^9^.

These characteristics may be accompanied by learning difficulties that may manifest as attention disorders and speech or language impediments. Early literature has also suggested a higher prevalence of autism spectrum disorder, attention-deficit/hyperactivity disorder, and schizophrenia; however, results are somewhat incomplete, as those studies did not take into account family history of mental illness or learning disabilities ^10^. Though these features are more apparent in the severe chromosomal aneuploidies (that is, 49, XXXXY), KS males may exhibit “language-based learning disabilities, decreased fine motor skills, and discrepancies between nonverbal and verbal cognitive abilities” in the sense that visuoperceptual and nonverbal skills appear to be strengths ^11^. In a cross-sectional study examining a prepubertal KS cohort, the presenting characteristic that ultimately led to diagnosis was developmental delay (11.6%) ^12^. Gropman and Samango-Sprouse ^13^ describe how young KS boys may have resultant academic difficulties in school, which broaches the topic of early diagnosis and whether testosterone replacement can prove advantageous. Although it is clear that androgens play a role in neurodevelopment, the progressive and more severe presentations of cognitive and motor delays seen with each additional X chromosome suggest a gene dosage effect of the X chromosome. In their double-blind randomized controlled trial, Ross *et al*. showed that 2 years of low-dose androgen treatment “did not have significant effects on most aspects of cognition (general cognition, verbal skills, working memory)” ^14^. Although the question of whether testosterone replacement would have a positive impact remains, early intervention with educational supports such as tutoring and counseling may be beneficial.

Diagnosis of the adolescent KS male can be more challenging. The degree of virilization is dependent upon the level of testicular testosterone production. The initial rise in testosterone levels during puberty may mirror that of a 46, XY male but typically plateau at low to low normal values. Although external secondary sexual characteristics may appear Tanner stage-appropriate as puberty progresses, reduced testicular volume is almost universally present. The average testicular volume for a KS postpubertal male is 2 to 5 mL ^15^. However, in a minority of cases, the degree of hypogonadism can be so severe that there may be minimal or no signs of pubertal development, prompting further workup. Testosterone supplementation to boost pubertal advancement may be of use in these cases.

KS discovered in adulthood is often under the circumstances of evaluation for primary infertility. Although 8% of the KS population may have detectable sperm in the ejaculate, the majority will have non-obstructive azoospermia ^16^. Typically, LH and FSH will be elevated, testosterone will be low normal, and semen volume will be adequate. Erectile function is not commonly impacted.

## What comorbidities can accompany Klinefelter syndrome?

As reviewed by Kanakis and Nieschlag ^17^ and Gravholt *et al*. ^18^, cardiovascular, cerebrovascular, and metabolic syndrome and other comorbidities are increased in patients with KS. Cardiovascular mortality is higher in this population and this is usually due to aortic valve anomalies, pulmonary embolism, peripheral vascular disease, and deep vein thrombosis ^19^. For example, as Zöller *et al*. detailed in their Swedish cohort study, the cumulative incidences of venous thromboembolism in men with KS were 8.6% at 50 years of age and 20.8% at 70 years of age ^20^. In efforts to explain this association, Valasek *et al*. ^21^ reported on a pediatric case in which the boy also had a type 1 protein C deficiency whereas Erkal *et al*. ^22^ did not find a difference in PAI-1 gene polymorphisms when comparing KS patients and cohorts. Finally, Glueck *et al*. caution that perhaps prothrombotic long-term testosterone replacement therapy (TRT) in conjunction with undiagnosed familial thrombophilia may be the combination that leads to thromboembolism in the patient with KS ^23^. They advocate for thrombophilia screening prior to initiation of TRT ^23^. Cardiac structural abnormalities include left ventricular diastolic dysfunction, mitral valve prolapse, and increased thickness of inner tunica of carotid arteries ^24^. Decreased exercise tolerance is also common. It is reasonable for practitioners to consider a diagnostic echocardiogram.

Cerebrovascular mortality may be secondary to subarachnoid hemorrhage, rupture of an intracranial saccular aneurysm, or a thrombotic event ^25^. The KS population is also noted to have a higher prevalence of metabolic syndrome and dyslipidemia. In one cross-sectional study, 47% of subjects with KS were noted to have metabolic syndrome, including increased total body fat, waist circumference, insulin resistance, triglycerides, and low-density lipoproteins ^26^.

Men with KS may have reduced bone mineral density in the lumbar spine, femoral neck, and total hip, but TRT does not ameliorate all of these findings ^27^. As Tahani *et al*. state, “bone loss in KS might, at least in part, be independent of hypogonadism” ^27^.

## Is there a predisposition to malignancy?

Although the overall incidence of malignancy is not higher in the KS population, there is an increase in multiple types of cancer, including breast cancer and extragonadal germ cell tumors (GCTs). However, the overall incidence is so low that routine screening of an asymptomatic KS male is not recommended.

According to Williams *et al*., 3% of GCTs diagnosed in males under the age of 19 occur in boys with a 47, XXY karyotype ^28^. Conversely, the risk of developing a GCT in KS patients of this age group is 1 in 4,000 (risk ratio of 18.8) compared with the normal 46, XY male population. In their study, the parent of origin of the supernumerary X chromosome was split equally between mother and father. Most GCTs in males with KS are mediastinal in location and teratomatous in histology. In fact, among KS males aged 14 to 19, GCTs are almost exclusively mediastinal. The exact biological etiology driving mediastinal GCT development is unknown at this point. These tumors may present at a young age as precocious puberty because of human chorionic gonadotropin secretion by the tumor cells. Older boys may present with chest pain or respiratory symptoms due to tumor location. Although today’s literature describes primarily pediatric cases of mediastinal GCTs in KS, adult males with KS have also been shown to develop these masses ^29^. Males with mediastinal GCTs should be screened for KS.

In her literature review of the data to date, Brinton concluded that the risk of breast cancer in the male with KS is 20- to 30-fold higher than expected and that there is a 60-fold increase in mortality ^30^. With the absolute incidence still being quite low, routine screening mammography is not a formal recommendation ^30^.

## What is the impact on spermatogenesis and implications for fertility?

KS is found to be the etiology of infertility in 10% of azoospermic males and accounts for up to 2% of infertility in the general male population ^31^. To give a brief embryologic overview, germ cells migrate to the genital ridges and populate the nascent seminiferous tubules. The Sertoli cells and Leydig cells form within and around the seminiferous tubules, respectively. Beginning just after birth, peaking in the 4th to 10th week after delivery and nadiring to prepubertal levels by 6 months of age, there is a surge in pituitary gonadotropins, also known as “mini puberty”, which stimulates the expansion and limited differentiation of the spermatogonial stem cell pool ^32^. The seminiferous tubules lengthen, testis volume enlarges, and Sertoli cell number increases ^33^. Cell cycle activity in the testes remains fairly quiescent until the initiation of puberty as a result of hypothalamic gonadotropin-releasing hormone (GnRH) stimulation of pituitary LH and FSH production and release. LH triggers intratesticular testosterone manufacture while FSH sparks Sertoli cell maturation and spermatogenic induction. In the KS male specifically, peripheral serum testosterone values rise but typically level off in the low normal range while the vast majority of 47, XXY spermatogonia become apoptotic as they fail to complete meiosis.

Classic puberty in the adolescent heralds the start of intratesticular testosterone production, necessary for spermatogenesis. Although testosterone levels do increase on schedule, males with KS experience a blunting of both Sertoli and Leydig cell function after the initiation of puberty. There is progressive apoptosis of the 47, XXY spermatogonia, ultimately resulting in azoospermia in adulthood ^34^. However, it cannot be simply that an inability to sequence through the meiotic cascade leads invariably to such rapid and dramatic cell senescence and demise, as we would then expect the same events to unfold in spermatocytic maturation arrest resulting from genetic causes. In this latter anomaly of spermatogenesis, viable spermatogonia and spermatocytes stack up like cars on a highway behind an impenetrable roadblock—remaining alive but prevented from progressing further. However, the presence of spermatozoa in about 50% of KS testes is best explained by postulating a low level of gonosomal mosaicism. Escaping death and proceeding through meiosis I and II, as well as spermiogenesis, are scattered 46, XY spermatogonia which, through a second quirk of nature, have lost the additional X chromosome, resorted to a normal diploid state, and come to reside in a seminiferous tubule here or a seminiferous tubule there. The distribution is most likely random, explaining the patternless and haphazard location within the tiny testes of spermatogenically healthy tubules.

Until the advent of assisted reproductive techniques, males with KS were considered to be sterile. However, with further understanding that these men may have focal areas of spermatogenesis within the testes, testicular sperm extraction (TESE) has become a mainstay treatment option for sperm harvesting and future reproduction ^35– 37^.

As described in the preceding paragraph, during mitotic renewal or differentiation of an occasional 47, XXY spermatogonial stem cell, the extra X chromosome may be lost because of anaphase lag ^38^. Subsequently, the resultant 46, XY daughter cells have the genetic opportunity to replicate. The extent of spermatogenesis in a male with KS appears then to depend on the presence and quantity of these 46, XY germ cells. Some argue that TESE procedures for sperm cryopreservation should be pursued during the adolescent period, or possibly earlier, prior to the progression of spermatogonial apoptosis. The opposite school of thought believes that TESE should be delayed until adulthood. As reviewed recently by Oates, many studies have examined this relationship between age at time of TESE and rate of successful surgical sperm retrieval (SSR) ^39^. Although some of the evidence is contradictory, the majority have found no difference in TESE outcomes among different age groups ^39, 40^. In fact, Franik *et al*. demonstrated that SSR was lower in patients below the age of 16 who underwent TESE ^41^.

To elaborate on and investigate spermatogenic potential in 47, XXY testes, van Saen *et al*. found that only 30% of peripubertal patients from 12 to 16 years had any germ cells on testicular biopsy, suggesting that germ cell loss begins well before puberty ^40^. The authors’ study beautifully addressed the timing of germ cell loss and the rate of TESE success in a number of different age ranges. They examined the testes of five fetuses with a 47, XXY karyotype and concluded that the number of germ cells identified “was not significantly different from controls”. There was very little fibrosis noted. However, Winge *et al*. observed that, in eight KS fetuses they studied, germ cell loss may already be occurring, specifically failure of gonocytes to differentiate into the slightly more advanced stage of pre-spermatogonia ^42^. The authors suggested an upregulation of X-chromosomal transcripts and enrichment of certain long non-coding RNAs (ncRNAs) as a possible etiologic explanation. Winge *et al*., in a further study, concluded that the mechanisms underlying germ cell loss may be different in the fetal, prepubertal, and adult stages and that aberrant maturation of Sertoli and Leydig cells may also be contributory ^43^.

In their study mentioned above, van Saen *et al*. also documented a paucity of detectable germ cells in prepubertal patients, no ongoing spermatogenesis in 20 biopsies from peripubertal boys, and sperm detection in only 1 out of 20 adolescent boys undergoing TESE. However, in the older adult patients with KS, TESE recovered fully formed spermatozoa in 48% with no difference noted between the various age groups (18–25, 26–30, 31–35, and >35 years), a finding supported by the meta-analysis by Corona *et al*. ^35^. To summarize, van Saen *et al*. concluded that “sperm recovery by TESE at early adolescent age does not appear to result in higher sperm retrieval efficiency compared to TESE at adult age”. Rives *et al*. also cautioned that “no predictive factors can actually demonstrate that early diagnosis of KS would allow increasing the chance of sperm retrieval” ^44^. Taking a slightly different and novel focus, Ragab *et al*. recently reported on the microsurgical TESE (mTESE) SSR in two overall groups of patients with KS: 31% in those with a history of unilateral or bilateral cryptorchidism and 38% in those with eutopic testes at birth—no statistical difference was seen. SSRs also did not differ between those KS men born with unilateral or bilateral undescended testes ^45^. Embedded within the excellent data of their study is the fact that, in the group of KS males with eutopic testes, the median age at which mTESE found sperm was comparable to the age at which no sperm were retrieved: 23 (range 15–26) and 20.5 (range 17–48), respectively ^45^.

In a discussion of preferred surgical techniques, a large systematic review by Corona *et al*., which compares techniques of TESE in patients with KS specifically, demonstrates similar sperm retrieval rates with both conventional TESE and mTESE ^35^. However, studies looking more generally at men with non-obstructive azoospermia do suggest that there is an increased sperm retrieval rate in mTESE ^46^. With regard to the ultimate goal of reproduction, the couple must proceed with intracytoplasmic sperm injection (ICSI) to use the spermatozoa that may be retrieved in either the freshly harvested or the frozen-thawed state. Recent studies report an average sperm retrieval rate per TESE procedure of 34% to 44% and an average live birth rate per ICSI cycle of 29% to 43% ^31, 35^. In their latest report, aptly titled “Is genetic fatherhood within reach for all azoospermic Klinefelter men?”, Vloeberghs *et al*. caution that a multiplicity of factors conspire to actually determine the ultimate chance of biological fatherhood, not just the probability of finding sperm on TESE or mTESE ^31^. To wit, only 14 out of 138 (10.1%) KS men and their partners starting treatment succeeded in having a live birth. Hormonal parameters have not yet been shown to predict the success of TESE ^31^.

Sex-chromosome aneuploidies are also fascinating from a purely genomic perspective. Although the supernumerary X chromosome undergoes inactivation, there are clearly genes that escape inactivation that are either directly or indirectly responsible for the KS phenotype. Via RNA sequencing (RNA-seq), Winge *et al*. demonstrated “211 differentially expressed transcripts in the fetal KS testis”, a significant enrichment of X-chromosomal transcripts and long ncRNAs ^42^. This suggested to them that the failure of gonocyte differentiation into pre-spermatogonia in the fetal testis led directly to the extremely poor (or absent) spermatogenesis in the adult KS testis. In their “companion” study on adult KS testes, they conclude that disturbed maturation of Sertoli and Leydig cells may further inhibit, and directly impair, spermatogenesis as well ^43^.

In their beautiful 2018 report, Skakkebæk *et al*. further defined the genetic underpinnings of the phenotypic findings in Klinefelter males ^47^. That is, they asked how the additional X chromosome is influencing form and function. They performed both genome-wide DNA methylation and genome-wide RNA-seq of peripheral blood leukocytes in 67 and 9 patients with KS, respectively. They had similar numbers of 46, XY male and 46, XX female controls for both groups. They built on previous work linking changes in the methylome and transcriptome of what we see in KS males ^48, 49^. They found 11 differentially methylated positions (DMPs) on the two X chromosomes when comparing KS and female controls (corresponding to eight genes) and 168 autosomal DMPs between KS and control males (corresponding to 85 genes). In their RNA-seq expression profiling studies, they found 31 differentially expressed genes between KS and male controls. Perhaps most importantly was the discovery that there were 23 differentially expressed autosomal and seven differentially expressed X-chromosomal ncRNA genes between the KS and control males. ncRNA genes may be involved in X inactivation as well as neurodevelopment and cognition ^50, 51^. Their conclusion forecasts future research and exploration efforts to causally link exact gene expression/regulation of the altered phenotype of KS males: “in conclusion, our results demonstrate a unique epigenetic and genetic landscape in KS involving both the X chromosome and the autosomal chromosomes, with few correlations between methylation values and gene expression” ^47^. Raznahan *et al*. added to this growing body of data by also reporting on sex-chromosome dosage effects on gene expression in hopes of teasing out why 47, XXY; 47, XXX; 47, XYY; 45, XO; and 48, XXYY are all phenotypically distinct ^52^.

A fair amount of focus has been placed on the role of androgen replacement therapy in KS, as testosterone levels are known to be low or low normal. Although this may be an important component of management with regard to pubertal advances, it will not have any positive effects on spermatogenesis or fertility. However, some studies suggest that TRT does not appear to impact sperm retrieval rates in patients with KS ^53, 54^. Some practitioners may employ replacement therapy just after the onset of puberty, typically in the form of gel, as compliance is higher ^54^. Aromatase inhibitors may be used in males with gynecomastia.

## Conclusions

As a genetic condition that affects 1 in 600 live male births, KS is being seen commonly enough that it is important for practitioners to understand the wide spectrum of issues and the multidisciplinary approach that is required to appropriately treat these patients. It is also essential to take note of distinct features that are known to be associated with KS, as this allows providers the opportunity to recognize undiagnosed cases. Along these lines, there is increasing momentum in genomic studies that are beginning to shed light on the differential gene expression that may explain the KS phenotypes and the variability within.

Despite ongoing investigations, there are still many essential questions, including whether hormone replacement therapy plays a beneficial role and what timing is appropriate for sperm retrieval for those interested in reproduction. The evidence to date suggests that hormone replacement therapy with testosterone may be valuable for peripubertal KS boys who appear to have delayed initiation of puberty and distinctly lack virilization. There is also a lack of data to support the advantage of TESE in the adolescent compared with the adult patient with KS. In fact, the counterargument, that optimal timing for TESE in the patient with KS is during early adulthood, is becoming more apparent.
